# Formula protein versus human milk protein and the effects on growth in preterm born infants

**DOI:** 10.1097/MCO.0000000000001084

**Published:** 2024-11-11

**Authors:** Jacqueline Muts, Britt J. van Keulen, Johannes B. van Goudoever, Chris H.P. van den Akker

**Affiliations:** a Department of Pediatrics, Emma Children's Hospital, Amsterdam UMC, University of Amsterdam; b Amsterdam Reproduction & Development Research Institute, Amsterdam UMC; c Department of Pediatrics – Neonatology, Emma Children's Hospital, Amsterdam UMC, University of Amsterdam, Amsterdam, The Netherlands

**Keywords:** donor human milk, mother's own milk, necrotizing enterocolitis, preterm infant formula, weight

## Abstract

**Purpose of review:**

This review aims to evaluate the latest available evidence on the differences between human milk proteins versus infant formula proteins and its effects on growth and development in preterm infants.

**Recent findings:**

High protein intake supports initial growth in preterm infants, although the long-term benefits remain unclear. Human milk requires adequate fortification to meet nutritional needs of preterm born infants. Formula feeding, with its higher protein content, may accelerate early weight gain but also increases the risk of necrotizing enterocolitis. Current evidence showed no significant advantages of human milk-derived fortifiers over bovine milk-derived fortifiers. Furthermore, studies published during the review period do not provide new evidence that alters the existing understanding of differences in neurodevelopmental outcomes between infants fed human milk and those fed formula.

**Summary:**

Both fortified human milk and preterm formula support growth in preterm infants, but human milk offers additional protective benefits, such as reducing the risk of necrotizing enterocolitis, making it the preferred option. Balancing immediate growth needs with potential long-term developmental outcomes remains crucial, highlighting the need for further research to determine the optimal protein intake for preterm infants.

## INTRODUCTION

Preterm infants face unique nutritional challenges due to their shortened intra-uterine growth period, resulting in high postnatal nutrient requirements and an increased risk of extra-uterine growth restriction. Adequate nutrition is crucial for supporting their growth and development, as postnatal growth failure in preterm infants is linked to poorer neurodevelopmental outcomes, while attempts at catch-up growth may increase the risk of metabolic syndrome later in life. Therefore, understanding and optimizing nutritional strategies for preterm infants is essential to support their long-term health and development.

Mother's own milk (MOM) is the preferred nutrition for all infants, including those born preterm [1^▪^]. When MOM is insufficient or unavailable for preterm infants, donor human milk (DHM) or preterm infant formula are the possible alternatives. The nutritional content of these milk sources differs substantially. Human milk alone does not meet the nutritional needs for protein, energy, and minerals of (very) preterm born infants in order to match intrauterine growth rates, necessitating fortification with additional macronutrients and minerals [1^▪^]. DHM is usually even lower in protein and fat content compared to MOM in the first postnatal weeks, as it is often donated at later lactational stages and because of nutrient losses in the preparation process. When DHM is the primary nutrition source, recent ESPGHAN guidelines therefore recommend higher than standard fortification levels as compared to fortification of MOM [1^▪^]. Conversely, preterm formulas typically contain higher protein, energy, and mineral levels than human milk but have poorer gastrointestinal tolerance and are associated with poorer outcomes, including an increased risk on developing necrotizing enterocolitis (NEC) [2,3^▪▪^].

Besides focusing on differences in macronutrient content between human and bovine milk sources, these milks also differ in bioactive components, like for example growth factors, immunoglobulins and lactoferrin, which play key roles in growth and immune function [4,5]. Additionally, processing methods used in infant formula factories and DHM banks can alter protein structures, potentially affecting their effectiveness in supporting infant growth and immunity [6].

This review of recently published literature since 2023 aims to explore the differences between human milk proteins and infant formula proteins and their impact on the growth and neurodevelopmental outcome of preterm infants. By examining the nutritional composition and the outcomes associated with different feeding strategies, we seek to provide latest insights into optimizing the care and feeding of preterm infants. 

**Box 1:**
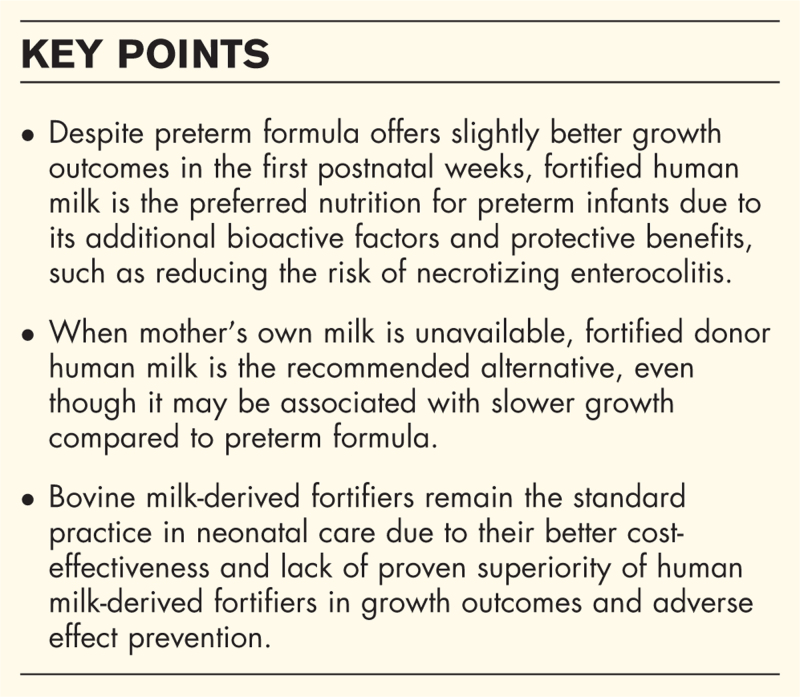
no caption available

## PROTEIN INTAKE AND PRETERM GROWTH

Preterm infants often face extra-uterine growth restriction due to cumulative deficiencies in protein and calorie intake. Given the essential role of protein in growth and development, these infants require an increased protein intake to support muscle and tissue development. A recent randomized controlled trial (RCT) conducted in Iran by Hemmati *et al.*[7^▪^] randomized 72 enterally fed preterm infants born <33 weeks’ gestation and compared conventional protein intake (3.5–4.0 g/kg/day) with high protein intake (4.0–4.8 g/kg/day) by adding a protein supplement (60% plant, 40% bovine) in both MOM and formula fed infants. Despite the study lasted on average less than two weeks, the study found significant improvements in weight gain (19.1 ± 7.2 versus 11.1 ± 5.3 g/kg/d, *P* < 0.001), head circumference gain (0.872 ± 0.262 versus 0.525 ± 0.210 cm/week, *P* < 0.001) and length gain (1.0 ± 0.35 versus 0.62 ± 0.20 cm/week, *P* < 0.001) in the high protein group. This study adds to the existing knowledge bank, demonstrating that increased protein intakes, independent of protein source (bovine or human), contribute positively to the growth outcomes in preterm infants during the first weeks of life.

Human milk of mothers who delivered preterm is known to have a slightly higher protein content in the first weeks of lactation compared to human milk of mothers who delivered at term. DHM is usually lower in protein content compared to MOM (term and preterm), as the milk is often donated at later lactation stages [8]. Two recent studies have compared preterm (donor) human milk with term donor human milk, and both studies found that preterm milk had a higher protein content than human milk of mothers who delivered at term [9^▪^,10^▪^]. Furthermore, the preterm milk was found to be more protein-dense with higher concentrations of sodium and chloride, while the fat content was similar [9^▪^]. Gialeli *et al.*[10^▪^] reported in an RCT (*n* = 120) that preterm born infants whose mothers had a shortfall of MOM and were therefore fed with preterm DHM in addition to MOM exhibited greater gain in weight and head circumference compared to preterm born infants fed with term DHM alongside MOM. These findings highlight the variability in the nutritional quality of different milk sources in the first postnatal weeks and the necessity of careful selection and supplementation to meet the nutritional needs of preterm infants.

A meta-analysis performed by Das *et al.*[11^▪^] assessed the effects of high protein (>3.5 g/kg/day) or low protein (<3.5 g/kg/day) intake (combination of parenteral and enteral intakes) during the first month of life on long-term outcomes. The study found that infants receiving higher protein intake showed slightly, though not significantly, higher weight z-scores (mean difference: 0.13 SD, *P* = 0.11), as well as increased fat mass, fat-free mass at discharge, and faster head circumference growth from birth to discharge. However, this early growth advantage did not continue into infancy, with infants who had initial higher protein intakes showing similar weight and length during early childhood. In the manuscript it is also stated that average head circumference was lower at toddler age in the high protein group, but upon our own review of the original data, it appears that some of the data in the corresponding forest plots were erroneously entered, so that in fact the head circumferences were larger on average. Besides, the validity of grouping parenteral and enteral nutrition together may be questioned [12]. Nonetheless, there remains an urgent need for also long-term follow-up data.

## MOTHER'S OWN MILK, DONOR HUMAN MILK AND FORMULA FEEDING: THE EFFECT ON GROWTH

Also during the review period, several studies have examined the impact of different milk sources, i.e. DHM versus preterm formula, on the growth and other clinical outcomes of preterm infants. The MILK trial by Colaizy *et al.* assessed the use of DHM compared to preterm formula in the near absence of MOM (with 53% receiving no maternal milk and the remaining 47% receiving minimal amounts for a median of 1 week or less) in an RCT in 483 extremely preterm infants [13^▪▪^]. DHM was fortified with bovine milk fortifiers according to the standard practice of each participating center. Extremely preterm born infants fed with DHM showed slightly lower weight gain compared with infants fed with preterm formula (22.3 ± 7.8 g/kg/day versus 24.6 ± 8.1 g/kg/day). However, it must be noted that both growth rates were faster than the average growth rate that is normally seen intrauterine, or during a NICU stay. Apart from the slightly slower growth rate, the most important finding in this trial was the significantly lower NEC incidence in the DHM group, highlighting the protective benefits of human milk.

In addition to the MILK trial, two other recent RCTs explored the effects of DHM versus formula on growth. However, in both of these trials, infants in both the control and intervention groups received substantial amounts of MOM alongside the supplement, complicating the ability to isolate the effects of DHM versus formula. The PREMFOOD pilot trial by Mills *et al.* found no persistent significant differences in growth measures or body composition as assessed by magnetic resonance imaging at term or six weeks post term (*n* = 103) [14]. In the pilot study by Pithia *et al.*, 32 late preterm neonates were randomized to receive, in case of a shortfall of MOM, either DHM or formula during the first week of life with the hypothesis DHM would increase breastfeeding rates during hospitalization [15]. The study found no differences in %MOM intake throughout hospitalization, but also not in weight, length and head circumferences at NICU discharge. In conclusion, no differences in growth were observed between infants fed with DHM compared to formula, as a supplement in case of a shortfall of MOM. These results should be interpreted with caution due to the small sample size in both pilot trials.

A recent Cochrane review update, which includes two of the RCTs which were discussed above, provides additional insights by comparing the effects of DHM versus preterm formula on the broader health outcomes of preterm infants [3^▪▪^]. The findings indicate that DHM instead of formula significantly reduces the risk of NEC by approximately 50%, though there is no clear indication it impacts late-onset sepsis incidence or overall mortality during hospitalization. Additionally, the review also showed that DHM potentially leads to slower weight gain until term equivalent age (mean difference −3.55 g/kg/day), although there was high heterogeneity in results. At last, there was no influence on weight or head circumference at 18 months postterm.

Apart from RCTs, also several cohort studies have recently examined the role of MOM, DHM, and formula on the growth of preterm infants. The first study of Kakaroukas *et al.* provides information on the growth and body composition in 107 moderate to late preterm infants who were either breast-fed, formula-fed, or mixed-fed between term equivalent age (TEA) and 3 months CA [16^▪^]. At TEA, the breast-fed group had the lowest mean lean mass, lean mass index, and fat mass when compared to the formula-fed group or mixed-fed groups. However, at 3 months CA, infants who were fully breast-fed had the highest gain in lean mass, lowest gain in fat mass, but absolute weights were no longer significantly different between groups. Another cohort study by Alyahya *et al.* compared in-hospital outcomes of very preterm infants (*n* = 907), stratified between those who had received mainly MOM (>90%), MOM supplemented with fortified DHM, or MOM supplemented with preterm formula during the first 14 days of life [17]. Weight and head circumference gains from birth to postnatal day 30 were similar between the three feeding groups. Important to note, though, is the lack of detailed information on the percentage of each milk type, making it unclear how much MOM, DHM, or formula was administered in each of the groups. Additionally, there were not enough subjects to perform the same comparison in the subgroup of extremely preterm infants, as only 11 extremely preterm infants received MOM supplemented with formula in this cohort.

The results of these studies highlight the complex interplay between protein intake from different milk sources and the outcomes of preterm infants. While human milk, particularly MOM, offers significant protective benefits, adequate multinutrient fortification which includes protein is essential in order to support growth sufficiently. Formula feeding may promote initial faster weight gain due to its often higher protein content, but will increase the risk of NEC. These findings highlight the need for tailored nutritional strategies to optimize growth and health outcomes in preterm infants.

## FORTIFICATION

Multinutrient fortification is a crucial strategy in enhancing the nutritional quality of milk for preterm infants, aiming to support their growth and development. Recently, more studies have assessed the use of human milk-derived fortifiers as an alternative to the commonly used bovine milk-derived fortifiers. The hypothesis is that avoiding bovine milk products and using exclusively human milk products could potentially reduce the risk of NEC and other adverse events.

An exclusively human milk-derived diet (MOM supplemented with DHM), including human milk-derived fortifiers, was randomly compared to a mixed fed diet of MOM with bovine milk-derived fortifiers and supplemented with preterm formula in 126 preterm infants (<30 weeks gestational age) by Embleton *et al.*[18^▪▪^]. The diet was maintained until 34 weeks PMA, and weight gain as well as morbidities were monitored until hospital discharge. The study found no significant differences in weight gain between the two groups from birth to discharge. Additionally, stool samples were analyzed to assess the gut microbiome, revealing no significant differences between the groups. The substantial intake of MOM in both groups may have contributed to the lack of observed differences in growth and microbiome composition.

In line with this, another recent RCT by Jensen *et al.*[19^▪▪^] investigated the effects of human milk-based fortifiers as compared to bovine milk-derived fortifiers on NEC, sepsis and mortality in extremely preterm infants fed exclusively with human milk (either MOM or DHM) (*n* = 228). The results did not show any significant differences in any of the studied clinical outcomes between the two fortifiers. The growth and neurodevelopmental outcomes of this trial have not been published yet but will be evaluated next. A retrospective cohort study by Lavassani *et al.*[20^▪^] compared the effects of human milk-derived and bovine milk-derived fortifiers on the growth parameters of preterm infants with very low birth weight. In this study, weight gain, head circumference, and length gain were assessed from birth to 4 and 8 weeks of age (*n* = 139 and *n* = 44, respectively). No statistically significant differences were observed between the groups, indicating that both types of fortifiers provided adequate protein and nutrients to support the growth of very low birth weight infants.

While fortification is essential for meeting the nutritional needs of preterm infants, current evidence does not show a significant advantage of human milk-derived fortifiers over bovine milk-derived fortifiers in terms of growth outcomes and the prevention of adverse outcomes. Since human milk-based multinutrient fortifiers are much more expensive [21], research has not shown superiority, and the ethics of for-profit human milk industry may even be questioned [22], ESPGHAN concluded that there is insufficient evidence to recommend the routine use of human milk-derived fortifiers [1^▪^]. Therefore, bovine milk-derived fortifiers remain the standard practice in most neonatal intensive care units due to their cost-effectiveness, although in some regions like in the United States their use is more common. Further research is necessary to conclusively determine whether any benefits exist with human milk-based fortifiers in the long term, especially considering that conditions like NEC are rare but extremely severe, requiring large-scale studies to detect any significant differences.

## NEURODEVELOPMENT

Neurodevelopmental outcomes are closely linked to the nutritional intake of preterm infants. Therefore, appropriate nutritional strategies are necessary to support both physical growth and cognitive development in preterm infants. The MILK trial by Colaizy *et al.*[13^▪▪^] with 483 extremely preterm infants, which is also discussed above, investigated whether DHM, compared with preterm formula, would result in better neurodevelopmental outcomes for extremely preterm infants. No significant differences were found in neurodevelopmental outcomes between the DHM and formula groups at 22 and 26 months CA. An analysis by Bando *et al.*[23^▪^] showed that while the macronutrient intakes during the first months were not associated with brain outcomes in forty preterm infants, a higher percentage of MOM intake was positively correlated with greater total and regional cortical gray matter volumes, which are linked to improved processing speed.

In the systematic review by Das *et al.*[11^▪^] on the effects of high protein (>3.5 g/kg/day) versus low protein (<3.5 g/kg/day) intake after birth on later neurodevelopmental outcomes in preterm children, authors found little to no difference in cognitive, language and motor scores during the first years of life between the high or low protein groups. This suggests that while higher protein intake may support immediate growth metrics, its influence on longer-term neurodevelopmental outcomes remains to be elucidated. Also, this systematic review did not discriminate between protein from human or bovine origin, and in fact even parenteral amino acids were included in the stratification. Brown *et al.*[24^▪^] reviewed several RCTs on amino acid and protein supplementation in preterm infants, concluding that higher enteral protein intake enhances short-term growth without adverse effects. However, the impact on neurodevelopment remains unclear, indicating the need for further research to establish whether there is a clear link between protein supplementation and long-term neurodevelopmental outcomes or not.

## CONCLUSION

Although preterm formula may result in slightly better growth outcomes in preterm infants, fortified human milk remains the preferred choice whenever available, as it has many additional protective benefits, including a reduced risk of NEC. If MOM is not available, DHM is the preferred alternative, as also recommended by organizations such as ESPGHAN. While MOM, and more frequently DHM, are sometimes associated with slower growth compared to preterm formula, this disadvantage can be mitigated through additional fortification. Fortifiers can be based on bovine milk products. Ongoing research is needed to further explore the long-term effects of different protein sources on preterm growth and body composition. The balance between immediate growth needs and potential long-term benefits must be carefully considered in nutritional strategies for preterm infants.

## Acknowledgements

*We thank Nerissa P. Denswil for her assistance in developing and executing the search strategy.*

### Financial support and sponsorship

*This research received no external funding.*

### Conflicts of interest

*J.M.: Nothing to disclose.*

*B.J.v.K.: Nothing to disclose.*

*J.B.v.G.: J.B.v.G. is the founder and director of the Dutch National Human Milk Bank and a member of the National Health Council. J.B.v.G. has been a member of the National Breastfeeding Council from March 2010 to March 2020. He received a grant to determine P.F.A.S. concentrations in human milk from The National Institute for Public Health and The Environment (RIVM) in 2024.*

*C.H.P.v.d.A.: C.H.P.v.d.A. reports receipt of speakers and consultancy honoraria from Nestlé Nutrition Institute, Nutricia Early Life Nutrition, and Baxter; used as research funds. He is vice-director of the Dutch National Human Milk Bank and a member National Breastfeeding Council since 2024.*

## REFERENCES AND RECOMMENDED READING

Papers of particular interest, published within the annual period of review, have been highlighted as:

▪ of special interest
▪▪ of outstanding interest
